# Reduced Translocation of Glyphosate and Dicamba in Combination Contributes to Poor Control of *Kochia scoparia*: Evidence of Herbicide Antagonism

**DOI:** 10.1038/s41598-018-23742-3

**Published:** 2018-03-28

**Authors:** Junjun Ou, Curtis R. Thompson, Phillip W. Stahlman, Nicholas Bloedow, Mithila Jugulam

**Affiliations:** 1 Department of Agronomy, Kansas State University, 2004 Throckmorton Plant Sciences Center, 1712 Claflin Road, Manhattan, KS 66506 USA; 2 Agricultural Research Center-Hays, Kansas State University, 1232 240th Avenue, Hays, KS 67601 USA; 3 Department of Statistics, Kansas State University, Dickens Hall 011, 1116 Mid-Campus Drive N, Manhattan, KS 66506 USA

## Abstract

*Kochia scoparia* is a troublesome weed across the Great Plains of North America. Glyphosate and dicamba have been used for decades to control *K. scoparia*. Due to extensive selection, glyphosate- and dicamba-resistant (GDR) *K. scoparia* have evolved in the USA. Herbicide mixtures are routinely used to improve weed control. Herbicide interactions if result in an antagonistic effect can significantly affect the management of weeds, such as *K. scoparia*. To uncover the interaction of glyphosate and dicamba when applied in combination in *K. scoparia* management the efficacies of different doses of glyphosate plus dicamba were evaluated under greenhouse and field conditions using GDR and a known glyphosate- and dicamba-susceptible (GDS) *K. scoparia*. The results of greenhouse and field studies suggest that the combination of glyphosate and dicamba application controlled GDS, but glyphosate alone provided a better control of GDR *K. scoparia* compared to glyphosate plus dicamba combinations. Furthermore, investigation of the basis of this response suggested glyphosate and dicamba interact antagonistically and consequently, the translocation of both herbicides was significantly reduced resulting in poor control of *K. scoparia*. Therefore, a combination of glyphosate plus dicamba may not be a viable option to control GDR *K. scoparia*.

## Introduction

Due to wide adoption of no-till agriculture, crop production in North American Great Plains is highly dependent on the use of herbicides^1^. However, because of the extensive and prolonged use of herbicides with the same site of action, a number of weed species evolved resistance to herbicides, which is one of the major threats to sustainable crop production. Additionally, rapid adoption of herbicide-resistant crops, is also contributing to the evolution of herbicide resistance in weeds due to lack of herbicide rotation, thereby increased selection pressure^2^.

*Kochia scoparia* (L.) Schrad. (*K. scoparia*), a member of Chenopodiaceae, was introduced to North America for ornamental purpose^3^. Soon after its introduction, *K. scoparia* has become highly invasive in crop fields and rangelands^3^. Because of its ability to tolerate drought, salinity, cold, and prolific seed production as well as tumbling mechanism of seed dispersal^3–6^, *K. scoparia* has turned out to be one of the worst weeds in North American Great Plains. Without timely management of *K. scoparia*, can cause huge yield loss in crops such as corn, sorghum, wheat, soybean, and sugarbeet^3,7^. In the last three decades, the situation has further exacerbated as a result of rapid and wide spread of resistance to acetolactate synthase-, photosystem II-, 5-enolpyruvylshikimate-3-phosphate synthase (EPSPS)- inhibitors, and synthetic auxins^8^. The management of *K. scoparia*, especially those populations that are herbicide-resistant has become an important component in cropping systems in North American Great Plains.

Combinations of multiple herbicides have been used to control a broader spectrum of weeds^9^, and to minimize the amount of herbicides applied^10,11^. More importantly, tank mixing of different herbicides has been recommended to delay the evolution of herbicide resistance in weed populations^12–14^. However, not all herbicides can be used in combinations, due to incompatibility or antagonism between certain herbicide chemical groups^15^. Combinations of glyphosate plus dicamba have been recommended as burndown application before planting no-till cotton to control horseweed (*Conyza canadensis* L.)^16^. In Kansas, glyphosate plus dicamba are usually sprayed together to manage wide spectrum of monocot and dicot weed species including *K. scoparia*, especially after evolution and spread of glyphosate resistance in weed populations since 2007^8^. However, inconsistent results when glyphosate plus dicamba combination were applied indicate that the interaction between these two herbicides can be species specific. For instance, O’Sullivan and O’Donovan^17^ reported antagonistic interaction between glyphosate and dicamba, resulting in decreased phytotoxicity of glyphosate on monocot crops such as wheat (*Triticum aestivum* L.), barley (*Hordeum vulgare* L.) and weeds, like wild oats (*Avena fatua* L.). Flint and Barrett^18^ reported that combinations of glyphosate plus dicamba could reduce the efficacy of glyphosate on johnsongrass (*Sorghum halepense* L.) due to reduced uptake and translocation. In contrast, Eubank *et al*.^19^ suggested addition of dicamba to glyphosate can increase control of horseweed from 70% to over 90%. However, the response of *K. scoparia* to glyphosate and dicamba combination is not known. When applied in combination, if these two herbicides exhibit an antagonistic interaction, this can result in poor control of *K. scoparia*, consequently, may accelerate the evolution of glyphosate and/or dicamba resistance because of exposure to less effective doses of these herbicides^20,21^, which in turn can weaken the herbicide options for the management of this weed. The glyphosate- and dicamba-resistant (GDR) *K. scoparia* populations have been reported and wide spread throughout the US Great Plains, including Kansas^8^. The significance of application of glyphosate and dicamba in combination on *K. scoparia* control is not known. Hence, it is important to investigate the interaction of glyphosate and dicamba on *K. scoparia*, to evaluate if use of these herbicides in combination help better control of this weed. Moreover, because growers are expected to rapidly embrace the new glyphosate- and dicamba-resistant crops, it is vital to understand the interaction of glyphosate and dicamba in *K. scoparia* to maintain the sustainability of the herbicide resistant crops in the *K. scoparia* infested regions. Therefore, this research was conducted with the following objectives: 1) test the efficacy of glyphosate plus dicamba combinations on GDR *K. scoparia* in greenhouse and field conditions; and 2) investigate the physiological interaction of glyphosate and dicamba in GDR *K. scoparia* using radioactive labelled herbicides, by comparing with a known glyphosate- and dicamba-susceptible (GDS) *K. scoparia*.

## Results

### Glyphosate- and dicamba- dose response of GDR and GDS *K. scoparia*

In all the experiments the label recommended doses of glyphosate and dicamba used were 840 and 560 g ae·ha^−1^, respectively. Analyses of herbicide dose response data (Table 1) suggested that the GDR *K. scoparia* were resistant to glyphosate at 840 g ae·ha^−1^. For instance, ED_50_ (effective dose for 50% control of *K. scoparia*) and GR_50_ (effective dose for 50% biomass reduction) of glyphosate for GDR *K. scoparia* were 978 and 835 g ae·ha^−1^ (Table 1), respectively, which were close to or higher than 840 g ae·ha^−1^. On the other hand, the values of ED_50_ and GR_50_ of glyphosate for GDS *K. scoparia* were significantly lower at 518 and 391 g ae·ha^−1^ (Table 1), respectively. Based on ED_50_ or GR_50_ estimates, the GDR *K. scoparia* was found to be twice more resistant to glyphosate than GDS (Table 1).

**Table 1:** Estimated values of ED_50_ and GR_50_ of glyphosate and dicamba in *K. scoparia* using the nonlinear regression analysis of four parameter log-logistic model*.

Herbicide	*K. scoparia*	ED_50_^#^	GR_50_^#^
		------g ae·ha^−1^------	
Glyphosate	GDR	978 (6)	835 (26)
	GDS	518 (38)	391 (28)
Resistance indices^+^		1.9 (0.2)	2.2 (0.3)
Dicamba	GDR	1259 (310)	2529 (438)
	GDS	72 (4)	106 (15)
Resistance indices^+^		18 (6)	25 (8)

^*^model: Y = C + (D−C)/(1 + exp[b(log(x) − log(I_50_))]); ED_50_ (effective dose for 50% control of *K. scoparia*) and GR_50_ (effective dose for 50% biomass reduction) values were estimated using the number of plants data and dry biomass data, respectively. ^#^Values in parenthesis are standard error. ^+^resistant level of GDR *K. scoparia* population comparing to GDS population using ED_50_ or GR_50_ values.

The results of dicamba dose-response suggested higher level of resistance to dicamba than glyphosate in GDR *K. scoparia*. The ED_50_ of dicamba for GDR and GDS *K. scoparia* were 1259 and 72 g ae·ha^−1^, respectively, whereas, GR_50_ estimates were 2529 and 106 g ae·ha^−1^, respectively (Table 1). These data suggest that the GDR *K. scoparia* is 20 times more resistant to dicamba than the GDS *K. scoparia*.

### GDR and GDS *K. scoparia* response to glyphosate plus dicamba combinations under greenhouse conditions

GDR and GDS *K. scoparia* response to herbicide combinations is presented as percentage of non-treated (%) in Table 2. The field recommended rate of dicamba (560 g ae·ha^−1^, Treatment (Trt) 1) and 1400 g ae·ha^−1^ (Trt 2, 2.5 times of field recommended rate) controlled 0 and 14% of GDR and 82% and 88% of GDS *K. scoparia*, respectively. When half of the recommended field rate of glyphosate (420 g ae·ha^−1^) was mixed with 350 g ae·ha^−1^ of dicamba (Table 2, Trt 3), it provided 22 and 47% of GDR and GDS *K. scoparia* control respectively. However, when 420 g ae·ha^−1^of glyphosate was mixed with 700 g ae·ha^−1^ of dicamba, it only provided 13 and 50% control of GRD and GDS *K. scoparia*, respectively.

**Table 2:** The treatments and efficacies (4 WAT) of glyphosate plus dicamba combinations on GDR and GDS. *K*. *scoparia* in greenhouse conditions.

Trt	Dicamba dose	Efficacy^*^			
		GDR		GDS	
	------g ae·ha^−1^------	------%------			
Glyphosate at 0 g ae·ha^-1^					
1	560	0 A		82(7) a	
2	1400	14(3) B		88(5) a	
Glyphosate at 420 g ae·ha^−1^					
3	350	22(8) A		47(8) a	
4	700	13(5) A		50(9) a	
Glyphosate at 840 g ae·ha^−1^					
5	0	45(3) A		95(1) a	
6	70	31(2) B		80(3) b	
7	140	30(3) B		82(2) bc	
8	280	30(5) B		91(3) ac	
9	560	43(3) A		87(2) ab	
Glyphosate at 1260 g ae·ha^−1^					
10	0	51(8) AB		96(1) a	
11	140	40(5) AB		92(3) ab	
12	280	57(9) A		96(1) a	
13	840	24(6) B		90(4) ab	
14	1400	48(7) AB		85(2) b	
Glyphosate at 2100 g ae·ha^−1^					
15	0	95(3) A		100 a	
16	140	56(5) C		97(2) ab	
17	280	59(5) C		92(3) ab	
18	700	93(4) AB		96(3) ab	
19	1400	79(2) C		91(1) b	
non-treated	0	0	0		0

^*^Means of visual injury (n = 20), and the values in parentheses are standard error. The values followed by different letters are significantly (*P-value* < 0.05) different among the treatments that contain the same dose of glyphosate within each population according to the Bonferroni’s multiple comparisons test.

In general, glyphosate alone without mixing with dicamba showed the best control of both GDR and GDS *K. scoparia*, compared to the combinations containing the same dose of glyphosate. For example, 840 g ae·ha^−1^ of glyphosate (Trt 5) had 45 and 95% control of GDR and GDS *K. scoparia*, respectively. It rendered more control of GDR *K. scoparia* than glyphosate and dicamba combinations (Trt 6, 7, and 8); and had similar control of Trt 9, which was mixed with 560 g ae·ha^−1^ of dicamba. Also, the 840 g ae·ha^−1^ of glyphosate (Trt 5) controlled GDS *K. scoparia* more effectively than Trt 6 and 7 (Table 2).

When Trt 10 to 14 were compared, 1260 g ae·ha^−1^ of glyphosate alone (Trt 10) rendered higher or similar control of the combinations that contain 140 to 1400 g ae·ha^−1^ of dicamba with the same amount of glyphosate. In the case of combinations with 2100 g ae·ha^−1^ of glyphosate, the results suggest that 2100 g ae·ha^−1^ of glyphosate (Trt 15) alone controlled the 95% of GDR *K. scoparia*, which is higher than Trt 16, 17, and 19 that were mixed with 140, 280 and 1400 g ae·ha^−1^ of dicamba, respectively. When 700 g ae·ha^−1^ of dicamba was mixed with 2100 g ae·ha^−1^ of glyphosate, the control of *K. scoparia* was similar to the application of 2100 g ae·ha^−1^ of glyphosate alone. However, all the combinations containing 2100 g ae·ha^−1^ of glyphosate rendered similar control of GDS *K. scoparia* except the Trt 19, which was mixed with 1400 g ae·ha^−1^ of dicamba and rendered only 91% control of GDS *K. scoparia*.

### GDR and GDS *K. scoparia* response to glyphosate plus dicamba combinations under field conditions

The results of *K. scoparia* control at 4 weeks after treatment (WAT) with combinations of glyphosate and dicamba are presented in Table 3. Similar to the results obtained under greenhouse conditions, the treatment 2.5 G (2.5 times of glyphosate at label recommended dose) controlled 98% of GDR *K. scoparia*, which is better than when treated with dicamba alone (e.g. 2.5D, 2.5 times of dicamba at label recommended dose). On the other hand, all the treatments with 2100 g ae·ha^−1^ of glyphosate, including the 2.5 G, 2.5 G + 1.25D (1.25 times of dicamba at label recommended dose), and 2.5 G + 2.5D, rendered 100% control of GDS *K. scoparia*. But, the treatment 2.5D that contained 1400 g ae·ha^−1^ of dicamba only provided 84% GDS *K. scoparia* control at 4 WAT, which is significantly less than the other treatments.

**Table 3:** The treatments and efficacies (4 WAT) of glyphosate plus dicamba combinations applied on GDR and GDS *K. scoparia* in field conditions.

Trt^*^	Herbicide doses		Efficacy^#^	
	Glyphosate	Dicamba	GDR	GDS
	------g ae·ha^−1^------		------%------	
2.5 G	2100	0	98(2) A	100 a
2.5 D	0	1400	18(5) B	84(5) b
2.5 G + 1.25 D	2100	700	83(6) C	100 a
2.5 G + 2.5 D	2100	1400	78(4) C	100 a
non-treated	0	0	0	0

^*^2.5 G, 2.5D, and 1.25D represents 2.5 times of glyphosate at label recommended dose (840 g ae·ha^−1^), 2.5 times of dicamba at label recommended dose (560 g ae·ha^−1^), and 1.25 times of dicamba at label recommended dose for *K. scoparia* control, respectively. ^#^Means of visual injury (n = 8), and the values in parentheses are standard error (n = 8). The values followed by different letters are significantly (*P-value* < 0.05) different among the four treatments within each population according to the Bonferroni’s multiple comparisons test.

### Absorption and translocation of [^14^C] glyphosate and [^14^C] glyphosate plus dicamba combination in GDR and GDS *K. scoparia*

**Figure 1:**
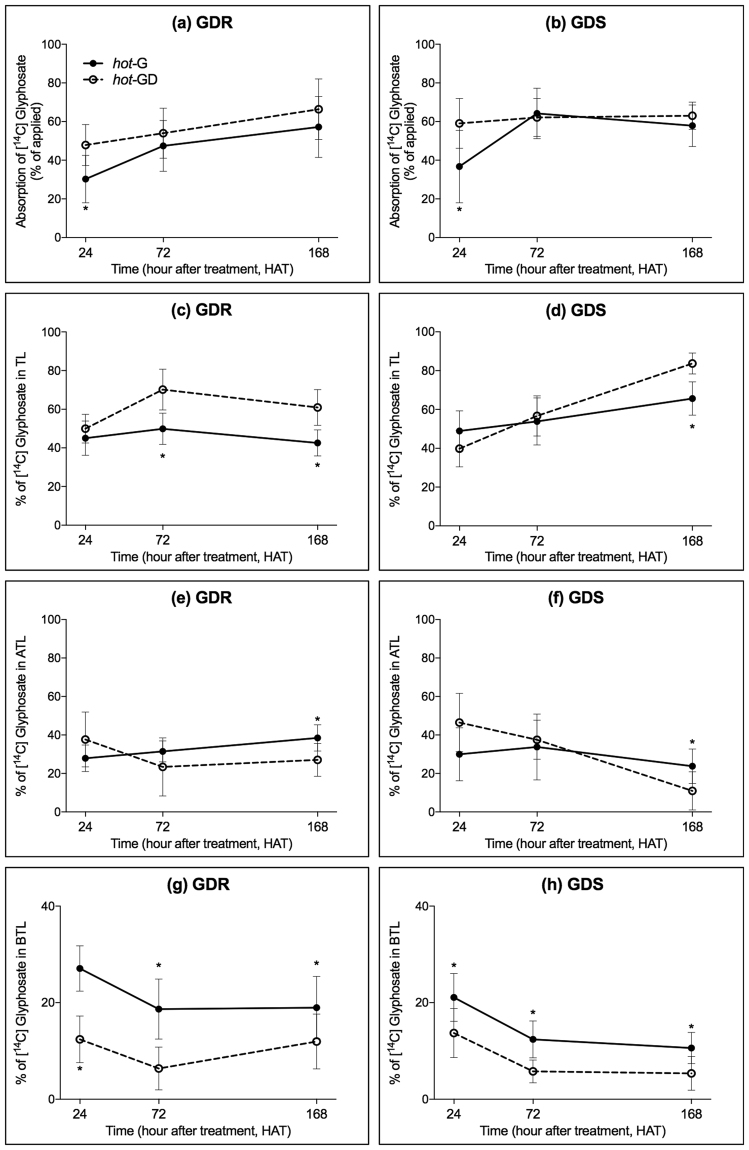
[^14^C] glyphosate absorption (**a** and **b**), retained in treated leaves (TL, **c** and **d**), translocated to above treated leave part (ATL, **e** and **f**) and below treated leave part (BTL, **g** and **h**) in glyphosate- and dicamba-resistant (GDR, **a**,**c**,**e**, and **g**), and glyphosate- and dicamba-susceptible (GDS, **b**,**d**,**f**, and **h**) *K. scoparia* when glyphosate alone (*hot*-G, solid line), and glyphosate plus dicamba combination (*hot*-GD, broken line) was applied. (*P-value < 0.05, which indicate the levels of significance at each time point for different herbicide treatments; error bars represent standard deviation, n = 8).

In both GDR and GDS *K. scoparia*, more [^14^C] glyphosate was absorbed when [^14^C] glyphosate was mixed with dicamba (*hot*-GD) than [^14^C] glyphosate was applied alone (*hot*-G) at 24 hours after treatment (HAT), e.g., 30.2 and 47.9% of [^14^C] glyphosate was absorbed at 24 HAT when *hot*-G and *hot*-GD was applied on GDR *K. scoparia*, respectively (Fig. 1a). However, there was no difference in herbicide absorption at 72 and 168 HAT between glyphosate or glyphosate plus dicamba combination in both GDR (Fig. 1a) and GDS (Fig. 1b) *K. scoparia*. In both GDR and GDS *K. scoparia*, the [^14^C] glyphosate translocation pattern suggest that more [^14^C] glyphosate was retained in treated leaves (TL) when *hot*-GD was applied than *hot*-G alone (Fig. 1c and d). The difference in translocation of glyphosate was observed at both 72 and 168 HAT in GDR *K. scoparia* (Fig. 1c), and it was also found at 168 HAT in GDS *K. scoparia* (Fig. 1d). This indicates less [^14^C] glyphosate was translocated away from TL when glyphosate was mixed with dicamba in both GDR and GDS *K. scoparia* (Fig. 1c and d). Also, less [^14^C] glyphosate was translocated to plant parts above-treated leaf (ATL) in both GDR (Fig. 1e) and GDS (Fig. 1f) *K. scoparia* at 168 HAT when glyphosate was mixed with dicamba than when was applied by itself. Especially, less translocation of [^14^C] glyphosate occurred in plant parts below-treated leaf (BTL) with *hot*-GD than *hot*-G. At all the time points tested, including 24, 72, and 168 HAT, significantly less [^14^C] glyphosate was translocated to BTL in both GDR (Fig. 1g) and GDS (Fig. 1h) *K. scoparia* with *hot*-GD than *hot*-G. Phosphor image analysis also confirmed these results, with less [^14^C] glyphosate translocated to shoots, leaves, and roots when glyphosate was mixed with dicamba in both GDR (Fig. 3b) and GDS (Fig. 3f) *K. scoparia*, compared to glyphosate alone in GDS (Fig. 3a) and GDR (Fig. 3e), respectively.

### Absorption and translocation of [^14^C] dicamba and glyphosate plus [^14^C] dicamba combination in GDR and GDS *K. scoparia*

**Figure 2:**
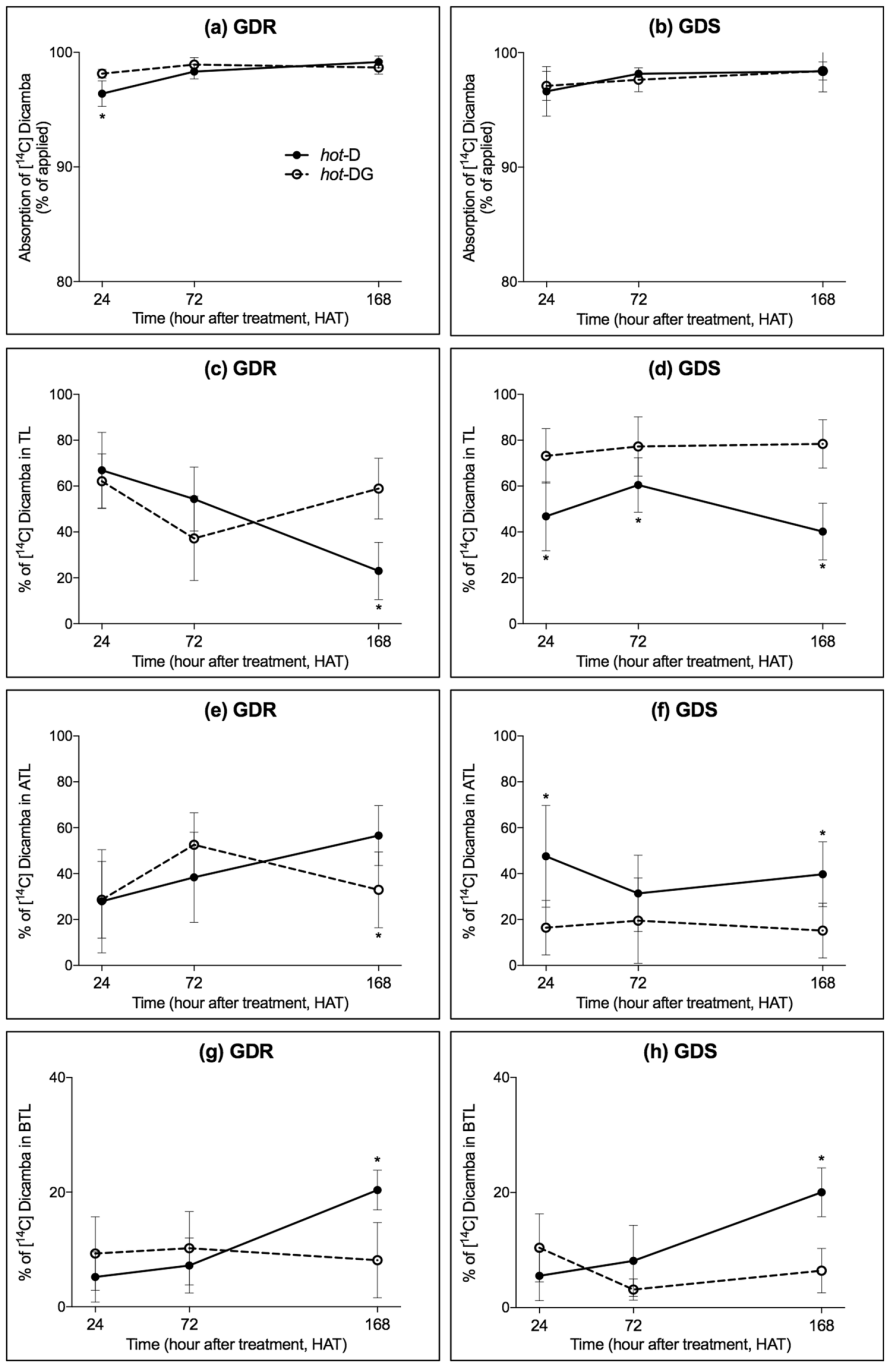
[^14^C] dicamba absorption (**a** and **b**), retained in treated leaves (TL, **c** and **d**), translocated to above treated leave part (ATL, **e** and **f**) and below treated leave part (BTL, **g** and **h**) in glyphosate- and dicamba-resistant (GDR, **a**,**c**,**e**, and **g**), and glyphosate- and dicamba-susceptible (GDS, **b**,**d**,**f**, and **h**) *K. scoparia* when dicamba alone (*hot*-D, solid line), and glyphosate plus dicamba combination (*hot*-DG, broken line) was applied. (*P-value < 0.05, which indicate the levels of significance within each time point for different herbicide treatments; error bars represent standard deviation, n = 8).

**Figure 3:**
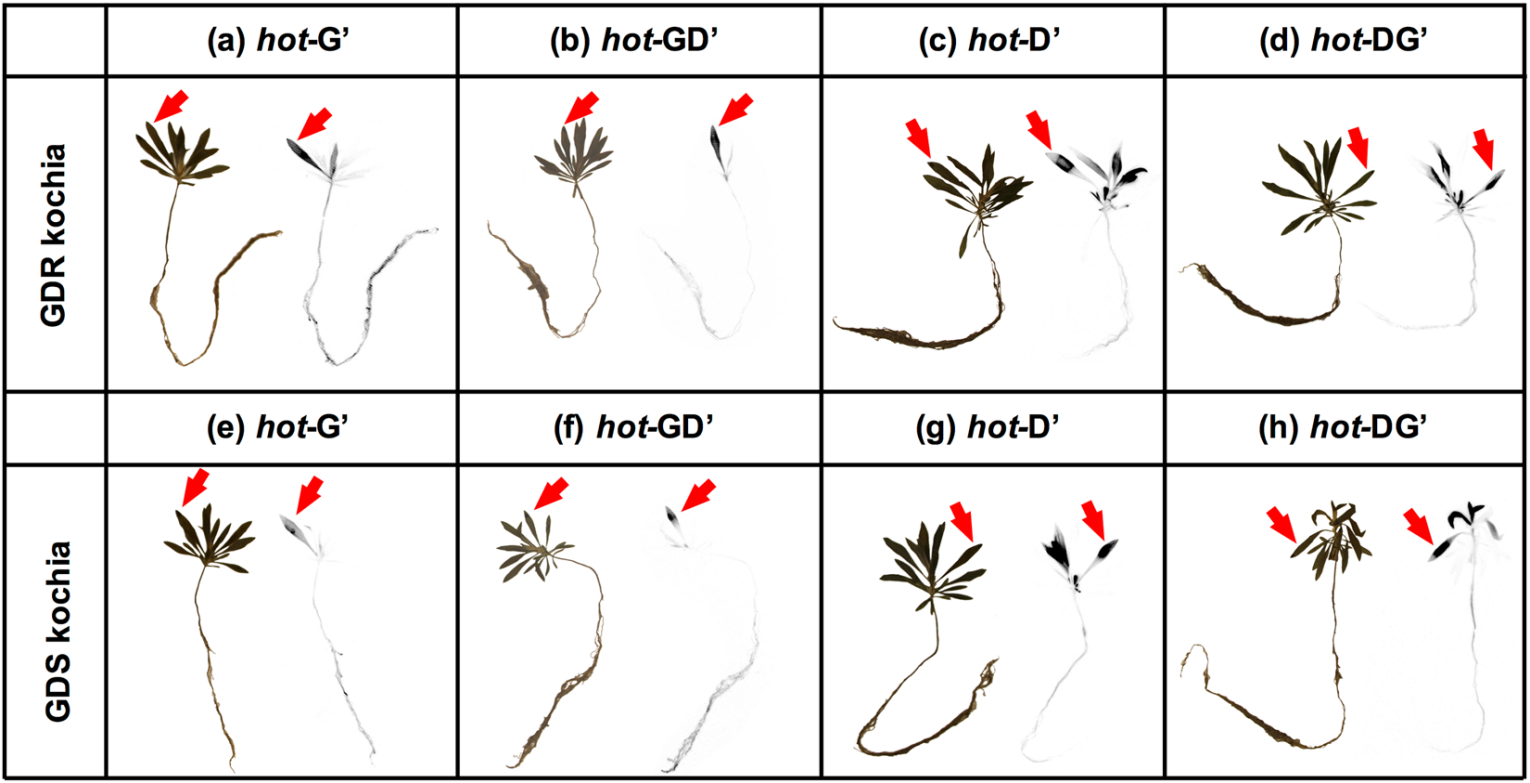
Phosphor images of [^14^C] glyphosate (*hot-*G’, **a** and **e**), [^14^C] glyphosate plus dicamba combination (*hot*-GD’, **b** and **f**), [^14^C] dicamba (*hot*-D’, **c** and **g**), and [^14^C] dicamba plus glyphosate combination (*hot*-DG’, **d** and **h**) in glyphosate- and dicamba-resistant (GDR, **a**–**d**) and glyphosate- and dicamba-susceptible (GDS, **e**–**h**) *K. scoparia* at 168 hours after treatment (HAT). Each plant is shown in RGB image (left) and phosphor image (right). In phosphor image, the darker color represents more radioactivity. Arrow points at the treated leaf on each plant where radioactive herbicide was applied. (Exposure times to phosphor screen were 44 hours for the *hot-*G’ (**a**,**e**) and *hot-*GD’ (**b**,**f**) treated plants, or 24 hours for the *hot-*D’ (**c**,**g**) and *hot-*DG’ (**d**,**h**) treated plants).

When glyphosate was mixed with [^14^C] dicamba (*hot*-DG), more [^14^C] dicamba was absorbed at 24 HAT in GDR *K. scoparia* (Fig. 2a) than when [^14^C] dicamba (*hot*-D) applied alone, but this difference was not observed at later time points tested (i.e. 72 and 168 HAT) in GDR (Fig. 2a) or in GDS *K. scoparia* (Fig. 2b). Translocation data indicate more [^14^C] dicamba was retained in TL at 168 HAT in GDR *K. scoparia* (Fig. 2c) for *hot*-DG than *hot*-D, and similar difference was observed in GDS *K. scoparia* at 24, 72, and 168 HAT (Fig. 2d). The translocation of dicamba to ATL or BTL also confirmed these differences. For instance, when dicamba was mixed with glyphosate (*hot*-DG), less [^14^C] dicamba was translocated to ATL at 168 HAT in GDR *K. scoparia* (Fig. 2e), and at 24 and 168 HAT in GDS *K. scoparia* (Fig. 2f). Also, less [^14^C] dicamba translocation to BTL at 168 HAT in both GDR (Fig. 2g) and GDS *K. scoparia* (Fig. 2h) was observed when dicamba and glyphosate were mixed. Furthermore, phosphor image analysis also supported that less [^14^C] dicamba was translocated to shoots when dicamba was mixed with glyphosate than was applied alone in both GDR (Fig. 3d vs. c) and GDS (Fig. 3h vs. g) *K. scoparia*.

## Discussion

The dose-response results confirmed that GDR *K. scoparia* is resistant to both glyphosate and dicamba, whereas GDS *K. scoparia* is susceptible to both glyphosate and dicamba. Furthermore, the GDR *K. scoparia* exhibited low level of resistance to glyphosate, whereas, resistance to dicamba was high relative to the GDS *K. scoparia*. Because of low level of resistance to glyphosate in GDR *K. scoparia*, increased glyphosate dose provided better control of GDR *K. scoparia* under both greenhouse and field conditions. In contrast increase in dicamba dose did not provide satisfactory control of GDR *K. scoparia* in any conditions tested. Growers tend to increase the herbicide dose to achieve maximum weed control. However, our results suggest that increase in herbicide dose may not always provide good weed control, rather increase the selection pressure, which facilitates evolution of resistance. These practices are not sustainable and should not be recommended since they may drive weed populations to evolve a higher level of resistance^22,23^.

Mixing herbicides with different sites/modes of action has been used widely to broaden the spectrum of weed control and delay the development of herbicide resistance^24,25^. In this research both under greenhouse and field conditions, we found that combinations of glyphosate plus dicamba had antagonistic effect on GDR and GDS *K. scoparia* control. When glyphosate was mixed with dicamba, the GDR *K. scoparia* control was significantly decreased compared to the same dose of glyphosate applied by itself (Tables 2 and 3). The GDS *K. scoparia* was controlled using most of the herbicide combinations tested, primarily because high doses of glyphosate and/or dicamba can mask the antagonistic effect of reduced translocation of these herbicides in GDS *K. scoparia*.

When applied in combination, the absorption of glyphosate and dicamba was enhanced at early hours than treated separately (Figs 1 and 2). Especially glyphosate absorption was increased in both GDR and GDS *K. scoparia* at 24 HAT (Fig. 1a and b); after that the difference in absorption was minimal, suggesting that mixing these two herbicides can accelerate absorption of both herbicides immediately after application. Accelerated absorption of dicamba possibly occurred because of the inclusion of adjuvant ammonium sulfate^26^. The rapid absorption of ammonium ions can reduce the apoplastic pH^27^, which can enhance dissociation of the dicamba diglycolamine salt (in Clarity^®^ formulation of dicamba) to form non-ionized dicamba acid and become more lipophilic. Once dicamba becomes more lipophilic it can be absorbed more quickly via waxy leaf cuticles, which are highly lipophilic^28,29^. However, this process could also increase the volatility of dicamba and upsurge the potential of dicamba drift due to presence of acid form of dicamba^30^, yet not completely absorbed by the plant. On the other hand, glyphosate absorption could have been enhanced by the adjuvants included in Clarity^®^ formulation, but additional study is needed to test this hypothesis.

Translocation of glyphosate was affected by dicamba regardless of time after application. When glyphosate was mixed with dicamba, less [^14^C] glyphosate was translocated and more was retained in treated leaves. This could occur as a result of rapid plant response to dicamba. As an auxinic herbicide, dicamba can cause rapid metabolic and physiological reactions within hours after application, which soon can lead to growth inhibition and reduction of transpiration and carbon assimilation^31^. Glyphosate is mainly transported via phloem^32^, which is highly dependent on the source-sink strength^33^. Therefore, due to weakened source upon dicamba application, the translocation of glyphosate may have been restricted compared to when glyphosate was applied alone. On the other side, reduced dicamba translocation was observed only at later time points when applied in combination. Glyphosate inhibits EPSPS enzyme and shuts down the shikimate pathway, which causes aromatic amino acid synthesis failure and stunts the growth of plants, and ultimately lead to plant death^34,35^. Within days, the glyphosate was translocated throughout the plant and shut down the shikimate pathway completely, soon after the carbon assimilation and phloem transport can cease. Therefore, the translocation of dicamba, which is also mainly facilitated by phloem^36,37^, would be significantly affected as a result of glyphosate-induced physiological alterations in plants.

In conclusion, though glyphosate plus dicamba combination is used to control a wide spectrum of monocot and dicot weeds in crops, this combination not necessarily is a good option to manage the stubborn weeds, such as *K. scoparia*, in North America Great Plains. Our results clearly suggest that glyphosate plus dicamba combination has significant antagonistic effect on both GDR and GDS *K. scoparia*, as a result of decreased translocation of these two herbicides resulting in reduced efficacy of both the herbicides. Therefore, if *K. scoparia* is the major issue in the field, glyphosate plus dicamba combination should not be recommended, especially when glyphosate and/or dicamba-resistant *K. scoparia* is present. Diversification of weed management tactics, such as inclusion of a third mode of action herbicide in the herbicide combination, or other non-chemical management practices such as tillage or cover crops are highly warranted to minimize the further development and spread of herbicide-resistant *K. scoparia*.

## Materials and Methods

In 2012, *K. scoparia* seed were collected from a field in Haskell County, Kansas (37°29′48.5″N, 100°46′53.0″W). *K. scoparia* plants generated from these seeds were self-pollinated by keeping the plants in isolation from other *K. scoparia* plants and upon maturity seed were harvested separately from ten plants. One hundred seedlings were generated separately from seed harvested from above 10 plants. When plants reached 10–12 cm height, 50 plants each were treated with a label recommended field rate of glyphosate (840 g ae·ha^−1^) or dicamba (560 g ae·ha^−1^). In response to glyphosate or dicmaba treatment, all the progeny of a single plant tested that were completely killed, these were selected as glyphosate- and dicamba-susceptible (GDS) *K. scoparia*. The remaining seed harvested from the same GDS mother plant was used in all experiments in this research. Likewise, all the progeny of single plant tested that survived glyphosate or dicamba treatment, were selected as glyphosate- and dicamba-resistant (GDR) *K. scoparia*. Also, the rest of the seed harvested from the same GDR mother plant was used in this research.

Greenhouse experiments were conducted in weed science greenhouse attached to the Department of Agronomy at Kansas State University, Manhattan, Kansas, United States. The following greenhouse conditions were maintained: 25/20 °C (day/night, d/n) temperatures, 60 ± 10% relative humidity, and 15/9 h d/n photoperiod supplemented with 120 μmol·m^−2^·s^−1^ illumination provided with sodium vapor lamps. The physiological studies were conducted in growth chambers maintained at following conditions: 25/15 °C d/n temperature, 60 ± 10% relative humidity, and 15/9 h d/n photoperiod, light was provided by incandescent and fluorescent bulbs delivering 750 µmol·m^−2^·s^−1^ photon flux at plant canopy level.

### Glyphosate- and dicamba-dose response of GDR and GDS *K. scoparia*

GDR and GDS *K. scoparia* seeds were germinated in trays (25 × 15 × 2.5 cm) filled with commercial potting mixture (Pro-Mix Potting-Mix, Premier Tech Horticulture, Ontario, CA). Individual seedlings at 6-leaf stage were transplanted into plastic pots (6.5 × 6.5 × 9 cm) containing the same type of soil and kept in the same greenhouse as above. When the *K. scoparia* seedlings were 10–12 cm height, they were treated with glyphosate (Roundup WeatherMax^®^, Monsanto Co., St. Louis, MO, USA) at 0, 52.5, 105, 210, 420, 840 (label recommended field, i.e. 1 × dose), 1680, and 3360 g ae·ha^−1^ with 2.5% (w/v) ammonium sulfate (AMS) or dicamba (Clarity^®^, BASF Corp., Florham Park, NJ, USA) without AMS at 0, 70, 140, 280, 560 (label recommended field, i.e. 1 × dose), 1120, 2240, 4480, and 8960 g ae·ha^−1^.

The above treatments were applied as follows. Herbicides were mixed according to the labels and applied using a bench-type sprayer (Research Track Sprayer, De Vries Manufacturing, Hollandale, MN, USA) equipped with a single moving even flat-fan nozzle tip (8002E TeeJet tip, Spraying Systems Co., Wheaton, IL, USA) delivering 187 L·ha^−1^ at 207 kPa in a single pass at 4.85 km·h^−1^. At four weeks after herbicide treatment (WAT), glyphosate- and dicamba-induced visual injury was rated based on composite visual estimation of growth inhibition, epinasty (downward curling of plant parts), necrosis, and plant vigor on a scale of 0 (no effect) to 100 (plant death). Plant were clipped off at soil level at 4 WAT and individual plants were placed in separate paper sacks. Dry biomass data was obtained by weighing after oven dried at 60 °C for 72 h.

### GDR and GDS *K. scoparia* response to glyphosate plus dicamba combinations under greenhouse conditions

GDR and GDS *K. scoparia* seedlings were produced as described above. When plants reached 10–12 cm height in the greenhouse, 19 combinations of low to high doses of glyphosate plus dicamba (Table 2) were applied (as described above) on both GDR and GDS *K. scoparia* to test their efficacy. At four WAT, the number of dead plants was recorded.

### GDR and GDS *K. scoparia* response to glyphosate plus dicamba combinations under field conditions

Field studies were conducted in 2015 and 2016 at Western Kansas Agricultural Research Center - Hays, Kansas, United States. To minimize the effect of herbicide residue, experimental plots were set in different fields next to each other in 2015 and 2016. The GPS coordinates of the field in 2015 and 2016 were 38°51′44.72″ N, 99°20′8.76″ W, and 38°51′44.72″ N, 99°19′59.34″ W, respectively.

In 2015, GDS and the GDR *K. scoparia* seeds were germinated in Planters Pride^TM^ plastic greenhouse kit (72 cells, The HC Companies, Middlefield, OH, USA) in the greenhouse. When the seedlings reached 3–4 cm, twenty plants of either GDS or GDR *K. scoparia* seedlings were transplanted by hand into each field plot of 3 × 3 m. The field was sprinkler irrigated daily. After the seedlings were recovered from transplantation and reached to 10–12 cm height, five treatments including 2100 g ae·ha^−1^ of glyphosate, 1400 g ae·ha^−1^ of dicamba, 2100 g ae·ha^−1^ of glyphosate mixed with 700 g ae·ha^−1^ of dicamba, 2100 g ae·ha^−1^ of glyphosate mixed with 1400 g ae·ha^−1^ of dicamba, and a non-treated control were used and designated as 2.5 G, 2.5D, 2.5 G + 1.25D, 2.5 G + 2.5D, and non-treated, respectively (Table 3), were applied using a CO_2_-pressured backpack sprayer with a 2.74 m boom that was equipped with six TTI110015 tip at 275 kPa with a spray volume of 140 L·ha^−1^ by walking at 4.8 km·h^−1^ approximately. Visual injury data (as described above) were collected at 1, 2, 3, and 4 WAT.

In 2016, the experiment was repeated using the same method as described above for in the year 2015, except GDS and GDR *K. scoparia* seeds were directly planted into the 3 m × 3 m plots, and hand weeding was implemented to remove other weeds.

### Absorption and translocation of [^14^C] glyphosate vs. [^14^C] glyphosate plus dicamba combination in GDR and GDS *K. scoparia*

In a previous research, we reported absorption or translocation of both [^14^C] glyphosate and [^14^C] dicamba in *K. scoparia* were not affected by spraying plants with formulated herbicides prior to application of [^14^C] labeled compounds^38^. In the same research, we also found that less than 5% of dicamba and glyphosate translocated to below ground tissue of *K. scoparia*^38^. Therefore, in this study, the *K. scoparia* plants were not treated with herbicide formulations prior to application of [^14^C] labeled dicamba or glyphosate, and also the radioactivity in below ground parts of *K. scoparia* was not tested.

One mL of [^14^C] glyphosate working solution (*hot-*G) with 0.33 kBq·µL^−1^ of radioactivity was prepared by mixing 93.6 µL of [phosphonomethyl-^14^C]-glyphosate (3.7 kBq·µL^−1^, specific activity: 2.04 kBq·µg^−1^, PerkinElmer, Inc., Boston, MA, USA), 20.5 µL of Roundup WeatherMax^®^ herbicide, 73.5 µL of AMS aqueous solution (34%, w/v) and 812.4 µL of water, which was equivalent to 2100 g of glyphosate in a carrier volume of 187 L water with 2.5% (w/v) of AMS. Another mL of [^14^C] glyphosate plus dicamba combination solution (*hot-*GD) with 0.33 kBq·µL^−1^ of radioactivity, which was equivalent to 2100 g of glyphosate and 1400 g of dicamba in a carrier volume of 187 L water with 2.5% (w/v) of AMS, was prepared by mixing 93.6 µL of [phosphonomethyl-^14^C]-glyphosate, 20.5 µL of Roundup WeatherMax^®^ herbicide, 15.6 µL of Clarity^®^ herbicide, 73.5 µL of AMS aqueous solution (34%, w/v), and 796.8 µL of water.

Radioactive herbicides were applied on GDR and GDS *K. scoparia* as follows. *K. scoparia* seedlings were grown in a growth chamber and when plants were 10–12 cm height, two newly expanded leaves were marked. Ten µL of *hot-*G or *hot-*GD solution (5 µL per leaf) was applied using Wiretrol^®^ (10 µL, Drummond Scientific Co., Broomall, PA, USA). Thirty minutes after herbicide application, plants were returned to the same growth chamber. Plant tissue was harvested at 24, 72 and 168 hours after treatment (HAT) and dissected into treated-leaves (TL), tissue above the treated leaves (ATL), and tissue below the treated leaves (BTL). TL were gently washed twice with 5 mL of 10% (v/v) aqueous ethanol solution with 0.5% of Tween-20 for one minute. Radioactivity in the rinsate was quantified using liquid scintillation spectrometry (LSS, Beckman Coulter LS6500 Multipurpose Scintillation Counter, Beckman Coulter, Inc. Brea, CA, USA) after adding 15 mL of Ecolite-(R) (MP Biomedicals, LLC. Santa Ana, CA, USA). Plant parts (TL, ATL, and BTL) were dried in oven at 60 °C for 72 h and combusted for three minutes with a biological oxidizer (OX-501, RJ Harvey Instrument, New York, NY, USA), the radioactivity in each plant part was quantified by LSS.

### Absorption and translocation of [^14^C] dicamba vs. glyphosate plus [^14^C] dicamba combination in GDR and GDS *K. scoparia*

The methods of application and sample collection of both [^14^C] dicamba (*hot-*D) and [^14^C] dicamba plus glyphosate (*hot-*DG) combination solution were the same as described above for glyphosate vs. glyphosate plus dicamba combination experiments, except that the 1 mL of *hot-*D working solution (equal to 1400 g of dicamba in a carrier volume of 187 L) with 0.33 kBq·µL^−1^ of radioactivity was obtained by mixing 29.3 µL of dicamba-(ring-UL-^14^C) ethanol solution (11.4 kBq·µL^−1^, specific activity: 2.87 kBq·µg^−1^, BASF Corp., Florham Park, NJ, USA), 15.4 µL of Clarity^®^ herbicide (BASF Corp., Florham Park, NJ, USA), 73.5 µL of AMS, and 881.8 µL of water; and the 1 mL of [^14^C] dicamba and glyphosate (*hot-*DG) combination solution (equal to 1400 g of dicamba and 2100 g of glyphosate in a carrier volume of 187 L) with 0.33 kBq·µL^−1^ of radioactivity was obtained by mixing 29.3 µL of dicamba-(ring-UL-^14^C) ethanol solution, 15.4 µL of Clarity^®^ herbicide, 20.8 µL of Roundup WeatherMax^®^ herbicide, 73.5 µL of AMS, and 881.8 µL of water.

### Phosphor image analysis of glyphosate/dicamba vs. glyphosate plus dicamba combination in GDR and GDS *K. scoparia*

Due to the nature of *K. scoparia* leaves, i.e. long and narrow, and to maximize the sensitivity of the phosphor image analysis, new working solutions of [^14^C] glyphosate, [^14^C] dicamba, [^14^C] glyphosate with formulated dicamba combination, and [^14^C] dicamba with formulated glyphosate combination containing 3.3 kBq·µL^−1^ of radioactivity, (denoted by *hot-*G’, *hot-*D’, *hot-*GD’, and *hot-*DG’, respectively), were prepared using the same method as described above.

GDR and GDS *K. scoparia* seeds were germinated in trays filled with the commercial potting mixture as described above. Individual seedlings 2 to 3 cm height were transplanted into plastic pots (6.5 × 6.5 × 9 cm) that filled with silica sand (Granusil® Handy Sand, Fairmount Santrol, Sugar Land, TX, USA) and rinsed in 1% (w/v) of Miricle-Gro water soluble All Purpose Plant Food (N:P:K = 24:8:16, Scotts Miracle-Gro Products Inc. Marysville, OH, USA) and kept in growth chamber. When the *K. scoparia* seedlings were 6–8 cm height (10–12 cm height plants were not selected, because the plants were taller to manuplate for phosphor image analysis), they were treated with 1 µL droplet of *hot-*G’, *hot-*D’, *hot-*GD’, and *hot-*DG’ on one newly expanded leaf. At 24, 72, and 168 HAT, *K. scoparia* plants were gently uprooted, and the roots were washed with water carefully. Then, the whole plant was washed twice with 10 mL of 10% (v/v) ethanol aqueous solution with 0.5% of Tween-20 for 1 minute, and then pressed using a handmade plant press^39^ and dried at 60 °C for 72 h. The pressed *K. scoparia* plants were exposed to BAS-IP MS 2040 E Multipurpose Standard Storage Phosphor Screen (GE Healthcare Life Sciences, Pittsburgh, PA, USA) for 44 h (the *hot-*G’ and *hot-*GD’ treated plants) or 24 h (the *hot-*D’ and *hot-*DG’ treated plants), and the screen was read using Bio-Rad molecular imager FX (Bio-Rad Laboratories, Inc. Hercules, CA, USA).

### Experimental design and data analysis

Split plot design was used in the experiment of glyphosate and dicamba dose response on GDR and GDS *K. scoparia*. *K. scoparia* population and herbicide dose were main- and subplot, respectively. Treatments were arranged in a factorial combination with GDR and GDS *K. scoparia* and different herbicide doses. No interaction between experimental runs was observed; hence, data from the repeated experiments were pooled prior to analysis. Then, visual injury and dry biomass data were subjected to non-linear regression analysis using four parameter log-logistic model^40^ in R (v.3.2.1, R Foundation for Statistical Computing, Vienna, Austria) with the *drc* package^41^.

1 $${\rm{Y}}={\rm{C}}+({\rm{D}}-{\rm{C}})/(1+\exp [{\rm{b}}(\mathrm{log}({\rm{x}})-\,\mathrm{log}({{\rm{I}}}_{50}))])$$

In Eq. 1, *Y* refers to the percentage of untreated, *C* and *D* are the lower limit and upper limit of the data, respectively, *b* is the slope, and *I*_50_ is the dose required for 50% response of visual injury or biomass reduction, which was used to estimate ED_50_ (effective dose for 50% control of *K. scoparia*) and GR_50_ (effective dose for 50% biomass reduction) values from the visual injury and dry biomass data, respectively.

Split plot experimental design was also used in greenhouse screening experiments and efficacy study of different glyphosate plus dicamba combinations in field conditions. *K. scoparia* population and rate of herbicide combination were the main- and subplot, respectively. Data from the repeated experiments were pooled prior to analysis due to no interaction between experimental runs was found. Two-way analysis of variance was performed in GraphPad Prism 7 (GraphPad Software, Inc., La Jolla, CA, USA) using Bonferroni’s multiple comparisons test (p-value < 0.05).

Randomized completely block design with a split plot and subsampling was used in the absorption and translocation of radioactive herbicides, where, *K. scoparia* population and herbicide (herbicide alone or combination) were the whole-plot treatment factors, the sampling times as split-plot factor, and the dissected plant parts as the subsampling factor. No interaction between experimental runs was found. Therefore, data of the total amount of absorption, percent of absorption, translocation amount, percent of translocation from repeated experiments were pooled prior to analysis and analyzed separately using SAS software version 9.4 (SAS Institute Inc., Cary, NC, USA) using the MIXED procedure.

Split plot experimental design was used in the phosphor imaging analysis, in which main- and subplot were *K. scoparia* population and sample harvesting time, respectively. Phosphor images were processed in Quantity One software 4.6.9 (Bio-Rad Laboratories, Inc. Hercules, CA, USA), and the plant photos were processed in GNU Image Manipulation Program 2.8.20 (GIMP development team, https://www.gimp.org).

At least four biological replicates (individual plants) of each treatment, dose or harvesting time were included for each experiment, and all the experiments were repeated twice in time.
